# Arrhythmias in Dengue: Beyond Prevalence, Toward Pathophysiology and Clinical Risk Stratification. Reply to Iqhrammullah, M.; Rampengan, D.D.C.H. Arrhythmias in Dengue: Moving Upstream from Electrocardiographic Findings to Cardio-Hemodynamic Dysfunction. Comment on “López-Delgado et al. Arrhythmias in Dengue: A Systematic Review and Meta-Analysis. *Pathogens* 2026, *15*, 497”

**DOI:** 10.3390/pathogens15070700

**Published:** 2026-07-02

**Authors:** Darío S. López-Delgado, Mathias S. Renteros-Ramirez, Joshua Emmanuel Arteaga-Bolaños, Harold E. Vásquez-Ucros, Kevin Alexander Burbano-Castro, Valentina Reina-Melo, Jessica Niebles-Blanco, Nancy Calzada-Gonzales, Lysien I. Zambrano, Valmore Bermudez, Alfonso J. Rodriguez-Morales

**Affiliations:** 1 Hospital Universitario Departamental de Nariño, Pasto 520002, Colombia; dario.lopezd@campusucc.edu.co; 2 Facultad de Medicina, Universidad Cooperativa de Colombia, Pasto 520002, Colombia; joshua.arteagab@campusucc.edu.co (J.E.A.-B.); haenvau@gmail.com (H.E.V.-U.); kevin.burbano@campusucc.edu.co (K.A.B.-C.); jesy_niebles@hotmail.com (J.N.-B.); 3 Facultad de Medicina, Universidad San Martin, Pasto 520002, Colombia; 170211231061@est.sanmartin.edu.co; 4 Facultad de Medicina, Universidad Peruana de Ciencias Aplicadas, Lima 15023, Peru; mathias.renteros@gmail.com; 5 Facultad de Medicina, Universidad Nacional Hermilio Valdizan, Huánuco 10004, Peru; calzadagonzalesnancy@gmail.com; 6 Department of Morphological Sciences, School of Medical, Sciences, Universidad Nacional Autónoma de Honduras, Tegucigalpa 11101, Honduras; lysien.zambrano@unah.edu.hn; 7 Centro de Investigaciones en Ciencias de la Vida, Facultad de Ciencias de la Salud, Universidad Simón Bolívar, Barranquilla 080002, Colombia; valmore.bermudez@unisimon.edu.co; 8 Faculty of Health Sciences, Universidad Cientifica del Sur, Lima 15074, Peru

We sincerely thank Iqhrammullah and Rampengan for their thoughtful and valuable Comment [1] on our systematic review and meta-analysis on arrhythmias in dengue [2]. We are grateful for their careful reading of our work and for recognizing its contribution to synthesizing the available evidence on the frequency and spectrum of electrocardiographic abnormalities in dengue. Their central point is highly relevant: arrhythmias in dengue should not be interpreted only as isolated electrical phenomena, but also within a broader cardio-hemodynamic and pathophysiological framework. Our meta-analysis showed that rhythm abnormalities are not uncommon in dengue and are predominantly represented by sinus bradycardia and sinus tachycardia [2]. The Comment appropriately emphasizes that these findings may often represent downstream manifestations of systemic illness rather than primary cardiac electrical disease.

We agree that our study should be regarded as a starting point for a deeper understanding of the relationship between dengue and cardiac rhythm disturbances. Dengue may affect the cardiovascular system through overlapping mechanisms, including plasma leakage, intravascular volume depletion, bleeding, fever, electrolyte abnormalities, autonomic imbalance, systemic inflammation, myocardial injury, and transient systolic or diastolic dysfunction. Prior evidence from Vietnamese patients has shown that severe dengue can be associated with transient systolic and diastolic myocardial impairment [3]. Similarly, studies in pediatric dengue shock syndrome suggest that preload reduction, decreased stroke volume, and altered systemic vascular resistance may all contribute to hemodynamic compromise [4]. These findings support the view that ECG abnormalities in dengue should be interpreted in light of the disease phase, severity, and clinical context, rather than as homogeneous arrhythmic endpoints. Future studies should therefore separate sinus rate abnormalities from clinically significant arrhythmias, including atrioventricular block, atrial fibrillation or flutter, supraventricular tachyarrhythmias, ventricular arrhythmias, QT abnormalities, and myocarditis-associated rhythm disturbances. They should also link ECG findings with warning signs, WHO severity classification, troponin, electrolytes, hematocrit dynamics, fluid balance, echocardiographic parameters, shock, inotrope requirement, intensive care admission, mortality, and post-acute cardiovascular outcomes.

The Singapore population-based cohort cited in the Comment is particularly important because it shifts the discussion from hospital-based ECG descriptions to population-level cardiovascular risk following dengue [5]. However, this approach should now be extended to Latin America, where dengue is endemic or hyperendemic and where viral circulation, host characteristics, comorbidities, vector ecology, access to care, and surveillance systems may differ substantially from those of Asian cohorts. Colombia is a relevant example. In Cauca, an endemic department with a severe dengue burden above national indicators, delayed consultation, hypotension, hepatomegaly, mucosal hemorrhage, platelet count below 100,000/mm^3^, and fluid accumulation are factors associated with severe dengue [6]. These findings reinforce the need to interpret arrhythmias within the broader clinical trajectory of dengue severity, because several of these predictors reflect hemodynamic compromise, vascular leakage, bleeding risk, and systemic involvement rather than isolated cardiac electrical disease. Therefore, Latin American populations should not only receive extrapolated evidence but also contribute directly to the global understanding of dengue-related cardiovascular disease.

In this context, the main contribution of our meta-analysis is not to provide a final explanation for dengue-associated arrhythmias, but to establish the magnitude of the problem and highlight the limitations of the available evidence. The next research step is to move from prevalence toward mechanism, prognosis, and clinical decision-making. Importantly, because the available evidence is predominantly observational and clinically heterogeneous, pooled estimates should be interpreted as measures of co-occurrence rather than proof that dengue directly causes specific arrhythmias. Residual confounding by disease severity, hemodynamic stress, fluid management, fever, electrolyte disturbances, and underlying cardiovascular conditions cannot be excluded. Prospective studies using standardized ECG definitions and serial cardio-hemodynamic assessment are therefore needed to distinguish direct myocardial or electrophysiological involvement from secondary manifestations of systemic dengue illness. A useful framework would consider dengue-associated arrhythmias as potential markers located along a continuum that includes immune activation, endothelial dysfunction, plasma leakage, altered preload, transient myocardial dysfunction, autonomic imbalance, systemic inflammatory stress, and, in selected cases, clinically significant cardiac involvement. Integrating ECG findings with hemodynamic assessment, myocardial biomarkers, imaging, disease phase, and patient-centered outcomes may improve risk stratification and help distinguish benign transient rhythm changes from clinically meaningful cardiovascular complications.

## Abbreviations

The following abbreviation is used in this manuscript:

ECG	Electrocardiogram
